# Health promotion behaviours of pregnant women and spiritual well‐being: Mediatory role of pregnancy stress, anxiety and coping ways

**DOI:** 10.1002/nop2.905

**Published:** 2021-05-03

**Authors:** Mohammad Chehrazi, Mahbobeh Faramarzi, Somayeh Abdollahi, Maria Esfandiari, Shiva Shafie rizi

**Affiliations:** ^1^ Department of Biostatistics and Epidemiology School of Public Health Babol University of Medical Sciences Babol Iran; ^2^ Social Determinants of Health Research Center Health Research Institute Babol University of Medical Sciences Babol Iran; ^3^ Student Research Committee Babol University of Medical Sciences Babol Iran

**Keywords:** anxiety, health promotion behaviours, pregnancy‐specific stress, prenatal coping, spiritual well‐being

## Abstract

**Aim:**

Little is known about the link between spiritual well‐being and health promotion behaviours in pregnant women. The study aimed to explore the direct and indirect effects of spirituality on health promotion behaviours with the mediatory roles of pregnancy stress, anxiety and coping ways.

**Design:**

Cross‐sectional.

**Methods:**

Two hundred women aged above 18 years completed Spiritual Well‐Being scale (SWBS), State‐Anxiety Inventory (SAI), Promoting Lifestyle Profile (HPLP), Prenatal Coping Inventory (Nu‐PCI) and Revised Prenatal Distress Questionnaire (NuPDQ).

**Results:**

Spirituality directly and negatively affected the state anxiety (*β* = −.41; *p* < .001) and NuPDQ (*β* = −.36; *p* < .001). Health promotion behaviours were negatively related to state anxiety (*β* = −.36; *p* < .001) and positively to planning‐preparation coping (*β* = .23; *p* = .001). Spirituality had a significant indirect effect on health promotion behaviours (*β* = .33; *p* < .001), mediated through its association with state anxiety and planning‐preparation coping. Thus, health professionals are proposed to consult pregnant women on the benefits of spirituality for improving healthy behaviours.

## 1 INTRODUCTION

Pregnancy is a unique event for every woman's life which is accompanied by psychological, social and physiological changes (Kazemi et al., 2018). This period requires physical and psychological adjustments for the women, which can affect pregnancy outcomes depending on their function (Vitorino et al., 2018). Meanwhile, the natural process of pregnancy can be disrupted by internal and external stressors (Pakzad et al., 2018). Stress may lead to adverse neonatal outcomes such as preterm labour, miscarriage and low birth weight (Faramarzi et al., 2019; Haghparast et al., 2016; Hasanjanzadeh & Faramarzi, 2017; Loomans et al., 2013). Thus, it is important to evaluate ways in which women cope with stress during pregnancy and to identify coping responses that decrease or, conversely, increase in prenatal emotional distress (Ibrahim et al., 2019). Coping responses are ways through which individuals can manage stress, and include active coping, avoidant coping and support coping (Faramarzi et al., 2016).

Spirituality covers components that have received little attention in coping theory in the context of pregnancy (Lucero et al., 2013), while being an important component of health and well‐being (Callister & Khalaf, 2010). Spirituality is divided into a framework of meaning and purpose, connectedness and values (Page et al., 2009). Lack of attention to the spiritual dimension of life and self‐knowledge may threaten mental health, growth and self‐actualization in humans (Dolatian et al., 2017). Recent evidence has reported the effect of spirituality on health outcomes through reducing the detrimental effects of stress on inducing inflammation (Shattuck & Muehlenbein, 2020).

Preparing for birth can be a profoundly spiritual experience, as mothers understand the miraculous nature of this phenomenon (Bélanger‐Lévesque et al., 2016). The birth is an ideal context in which the spiritual dimension of women's lives can be acknowledged (Callister & Khalaf, 2010). According to studies on maternal health during pregnancy, spirituality has preventive effects on stress during pregnancy (Dolatian et al., 2017; Lucero et al., 2013). Feelings of belonging to a superior power and faith in God as well as spiritual support under stressful life conditions help religious people enjoy better mental health and suffer less from life's problems (Pakzad et al., 2018). A study confirmed the impact of spirituality on specific pregnancy stress reduction (Dolatian et al., 2017). Another study indicated that greater spirituality is associated with fewer depressive symptoms in pregnant women (Vasegh et al., 2012). In contrast, negative use of spirituality, albeit rare, is associated with poor outcomes, including high prevalence of anxiety, stress, depressive symptoms, impaired quality of life and dissatisfaction with health status (Kang & You, 2018).

Health promotion behaviours are any kind of conscious planning and functioning, which aim to prevent disease, improve health, boost productivity, prevent negative consequences and achieve individual self‐actualization (Kazemi et al., 2018; Lin et al., 2009). The pregnancy period is very important as the maternal behaviours affect the childbirth outcomes as well as quality of life of both the mother and child (Kazemi & Hajian, 2018). Research has emphasized that addition of spiritual resources to healthy lifestyle behaviours may be important to maternal and child health (Motahari Tab ari et al., 2019). A research indicated that increased spirituality is associated with diminished likelihood of alcohol use, smoking, marijuana use and better maternal nutrition during pregnancy (Cyphers et al., 2017). A review reported that people who are more spiritual have better mental health and healthy behaviours compared to those with less spirituality (Koenig, 2012).

Although previous studies have emphasized the role of spirituality in improving physical and mental health (Callister & Khalaf, 2010; Dolatian et al., 2017; Shattuck & Muehlenbein, 2020), there is little information about spirituality and health promotion behaviours in pregnant women (Cyphers et al., 2017; Dolatian et al., 2017). This study developed a theatrical pathway model to test the relationship between spirituality and health promotion behaviours among pregnant women and whether state anxiety, pregnancy stress and coping strategies mediate the relationship. The conceptual model of the indirect effect of spirituality on health promotion behaviours was proposed according to previous evidence. Shattuck and Muehlenbein (2020) proposed that spirituality is associated with lower levels of anxiety and less perceived stress. Also, researchers have found that anxiety negatively predicted healthy behaviours of pregnant women (Omidvar et al., 2018). Further, spirituality may improve the healthy behaviours of pregnant women by promoting adaptive copings (Faramarzi et al., 2016). Another research reported that spirituality is correlated with problem‐solving strategies in pregnant women (Faramarzi et al., 2017).

Little is also known about the structural equations through which the psychological factors influence the healthy behaviours through spirituality. The current study addresses the existing gap in research on the interaction between spirituality and health promotion behaviours based on testing a model which examines the effect of spirituality on health promotion behaviours through the mediating role of stress and coping ways. To the authors' knowledge, this is the first study investigating the direct and indirect effects of spirituality, stress and coping ways on health promotion behaviours in pregnant women. Indeed, the aim of the study has been to explore the direct and indirect effects of spirituality on health promotion behaviours with the mediatory roles of pregnancy stress, anxiety and coping ways.

## 2 MATERIALS AND METHODS

This research study initiated from June to October 2018 after approval of the ethics committee of Babol University of Medical Sciences (MUBABOL.HRI.REC.1396.62). Further, all participants signed a written informed consent prior to participating in this study. Two public prenatal care clinics were randomly selected. The pregnant women referring to these clinics were sampled to participate voluntarily in the study. Two hundred pregnant women aged 18 years or older with gestational age of at least 12 weeks were eligible for the present study. Those who had not passed 5 years of school or could not complete the self‐report questionnaires were excluded from the study. Two trained midwives separately checked the study inclusion criteria in each clinic and asked the demographic information of eligible participants and gave them questionnaires to be completed. All participants completed five questionnaires including Spiritual Well‐Being scale (SWBS), State‐Anxiety Inventory (SAI), Promoting Lifestyle Profile (HPLP), Prenatal Coping Inventory (Nu‐PCI) and Revised Prenatal Distress Questionnaire (NuPDQ).

### 2.1 Measurements

#### 2.1.1 Spiritual well‐being scale (SWBS)

This scale was designed by Paloutzian and Ellison (1991) to measure one's perception of the spiritual quality of life and life satisfaction. It contains 20 items with two subscales: existential well‐being (EWB) and religious well‐being (RWB), with each subscale containing 10 items. The SWBS contains some positive and some negative items. Each item is scored on a six‐point Likert scale, ranging from 1–6. Scoring is ordered by a 6‐point Likert scale as follows: (1) strongly disagree, (2) moderately disagree, (3) disagree, (4) agree, (5) moderately agree and (6) strongly agree. Negatively worded items (item numbers 1, 2, 5, 6, 9, 12, 13, 16 and 18) have reversed scores such that higher scores represent a greater level of well‐being (Paloutzian & Ellison, 1991). In the study, we used the validated Persian version. Persian SWBS has good reliability (*α* = .85) and internal consistency (*α* = .97) (Abhari et al., 2018).

#### 2.1.2 Health promoting lifestyle profile (HPLP‐II)

Walker et al. (1987) developed HPLP‐II (Tanjani et al., 2016) to determine the health promotion behaviours. HPLP‐II contains 52 questions with six aspects of health‐promoting behaviours including nutrition (nine items), physical activity (eight items), spiritual growth (nine items), health responsibility (nine items), stress management (eight items) and interpersonal relations (nine items). Items are scored based on a four‐point Likert scale from 1 (never) to 4 (always). The scores range from 52–208. Cronbach's alpha of the revised HPLP‐II was reported as 0.87. Also, confirmatory factor analysis demonstrated the fit of the six component structure of the subscales (Tanjani et al., 2016). We used the validated Persian HPLP‐II version. The Cronbach's alpha of the Iranian version of HPLP‐II was reported as 0.78 (Tanjani et al., 2016).

#### 2.1.3 State‐anxiety inventory (SAI)

The SAI, a subscale of the STAI, measures state anxiety. The state anxiety scale consists of 20 questions that determine how the respondents ‘feel right now’. Of the 20 statements, 10 capture anxiety‐present items and the rest cover anxiety‐absent items. Each item is scored on a four‐point scale, ranging from 1 (not at all) to 4 (almost always). The total score of the SAI can ranges from 20–80. This scale has high validity (*α* = .89) and reliability (ICC = 0.86). We used the validated Persian SAI version. The Cronbach's alpha of the Persian version of SAI was reported as 0.90 (Panahi‐Shahri, 2002).

#### 2.1.4 Revised prenatal coping inventory (Nu‐PCI)

The Nu‐PCI is a revised version of the 36‐ item PCI developed by Hamilton and Lobel (2008). A specific self‐report instrument focuses on the coping style of pregnant women during the prenatal period. The Nu‐PCI subscales include planning‐preparation, spiritual‐positive coping and avoidance. Respondents report how often they would use different kinds of coping in the past month on a scale from 0 (never) to 4 (very often). The higher the mean subscale score, the more frequently that coping style was used. The Cronbach's alpha for the planning‐preparation subscale in early, mid and late pregnancy was 0.82, 0.85 and 0.86, respectively. The Cronbach's alpha for the avoidance subscale ranged from 0.77–0.80 during pregnancy. The Cronbach's alpha for the spiritual‐positive subscale varied from 0.73–0.78 over the three trimesters of pregnancy (Hamilton & Lobel, 2008). We applied the validated Persian Nu‐PCI version. The internal consistency of the Persian scale lied within an acceptable range (*α* = .89–.97). The reliability of the Persian Nu‐PCI and subscales with test‐retest coefficients was 0.98–0.99 (Faramarzi et al., 2017).

#### 2.1.5 Revised prenatal distress questionnaire (NuPDQ)

The scale is a revised version of the 12‐items PDQ (1999) (Yali & Lobel, 1999). It contains 17 items assessing the distress associated with pregnancy‐specific concerns, including bodily changes, physical symptoms, foetal health, labour and delivery. Responses are given on a three‐point scale ranging from 0 (not at all) to 2 (very much) (Lobel et al., 2008). NuPDQ has a Cronbach's alpha of 0.71 for internal consistency and a Cronbach's alpha of 0.56 to 0.72 for ICC (Lobel et al., 2008). We used the validated Persian version of NuPDQ. Cronbach's alpha of reliability of NuPDQ was 0.96 (Esfandiari et al., 2020).

### 2.2 Partial least square structural equation (PLS‐SEM) modelling

**FIGURE 1:**
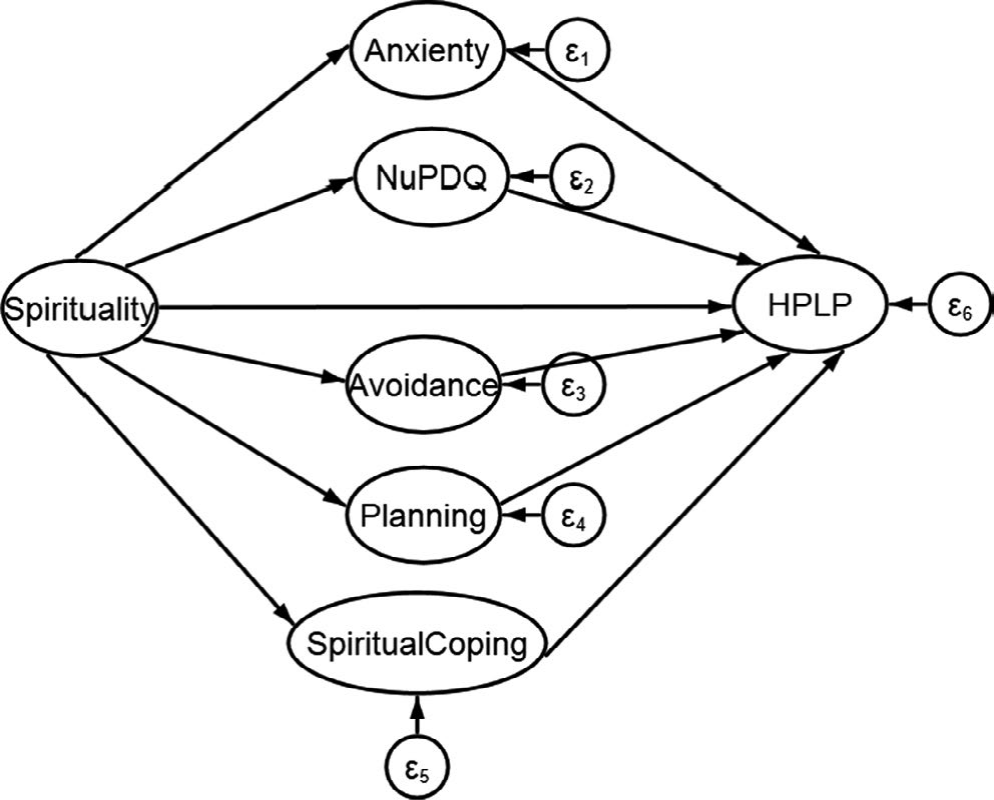
Path diagram of the Conceptual model depicting direct and indirect association between spirituality and Health Promoting Lifestyle Profile (HPLP)

A PLS‐SEM model was applied to determine whether spirituality can affect the health promotion behaviours through anxiety, pregnancy‐specific distress and coping subscales ‐ as mediator variables ‐ and whether there exists a causal relationship between them. The conceptual model is depicted in Figure 1 as expressed by the structural equation modelling.

### 2.3 PLS‐SEM model assessment

In order to assess the PLS‐SEM model fitting, two steps were taken. First, internal consistency reliability was measured using rhoA proposed by Dijkstra and Henseler (2015). The second step was to assess the convergent validity of each construct measured by the average variance extracted (AVE) computed for each construct (latent variable). The variance inflation factor (VIF) was employed to evaluate collinearity of the latent variables in the constructed model. We also computed pairwise Pearson's correlation between the constructs. STATA software Version 15 (STATA Corp) was used to run PLS‐SEM and other relevant analyses. A *p*‐value < .05 were considered statistically significant.

## 3 RESULTS

### 3.1 Sample characteristics and correlations

The investigated sample consisted of 200 eligible pregnant women (184 of the pregnant women [92%] were housewives). The participants' age ranged from 17–44 years (Mean = 27.5; *SD* = 5.34). A total of 49 (24.5%) participants stated university degree as their highest educational level, 108 (54.5%) a diploma degree, 39 (19.5%) a high school degree and 4 (2%) participants declared a completed primary school degree as their highest educational level. The mean gestational age at the time of filling the scales was 23.98 weeks (*SD* = 6.61, Table 1).

**TABLE 1:** Demographic characteristics of study participants *N* = 200

Variable	Mean ± *SD* Frequency (%)
Age	27.5 ± 5.34
Gestational age	23.98 ± 6.61
Educational level	
Primary	4 (2%)
High school	39 (19.5%)
Diploma	108 (54.5%)
University‐educated	49 (24.5%)
Job	
Housekeeper	184 (92%)
Employed	16 (8%)

Descriptive results of the constructs including mean and standard deviation (*SD*) as well as the pairwise correlation coefficients between them are provided in Table 2. Correlations between the examined constructs indicated that the total spirituality score was significantly negatively correlated with state anxiety and pregnancy stress(*p* < .001). On the other hand, spirituality was positively linked to planning‐preparation, avoidance, spiritual‐positive coping and health promotion behaviours (all *p* < .001) (see Table 2). In addition, state anxiety was negatively linked to health promotion behaviours (*p* < .001), while planning‐preparation was positively related to health promotion behaviours (*p* < .001).

**TABLE 2:** Pairwise Pearson's correlation coefficients between constructs and their descriptive statistics (Mean and *SD*)

Construct	1	2	3	4	5	6	7
1. SWBS	1						
2. State anxiety	−0.41*	1					
3. NuPDQ^a^	−0.36*	0.47*	1				
4. Planning	0.35*	−0.29*	0.0032	1			
5. Avoidance	0.46*	−0.40*	−0.24*	0.60*	1		
6. Spiritual‐positive coping	0.48*	−0.34*	−0.21*	0.54*	0.58*	1	
7. HPLP	0.45*	−0.59*	−0.34*	0.47*	0.48*	0.45*	1
Mean	101.5	39.23	11.6	41	27.07	18.21	135.06
*SD*	10.59	8.32	4.82	10.56	7.23	3.34	22.49

Abbreviations: *SD*, Standard deviation; SWBS, Spiritual Well‐Being Scale.

^a^ Revised Prenatal Distress Questionnaire.

* *p* < .001.

### 3.2 PLS‐SEM model fit assessment

The internal consistency rhoA for constructs is shown in Table 3. All values of rhoA were within an acceptable range (0.47–0.95). Further, the average variance extracted (AVE) for all constructs was below 0.5, suggesting that the construct would explain less than 50% of the variance of the items making up the construct (Table 3). Finally, all VIF values were lower than 3, suggesting that collinearity issues in the construct model were not considerable.

**TABLE 3:** Results of PLS‐SEM model fitting internal consistency, convergent and discriminant validity

Fitting Index	SWBS	State anxiety	NuPDQ^a^	Planning	Avoidance	Spiritual‐positive coping	HPLP
RhoA	0.86	0.90	0.47	0.88	0.64	0.68	0.95
AVE	0.27	0.32	0.12	0.33	0.14	0.40	0.27

Abbreviations: HPLP, Health Promoting Lifestyle Profile; SWBS, Spiritual Well‐Being Scale.

^a^ Revised Prenatal Distress Questionnaire.

### 3.3 Effect of spirituality on health promotion behaviours

The PLS‐SEM results provided us with the necessary information to test the hypotheses related to the direct and mediational effects of variables.

The causal model (displayed in Table 4) revealed that spirituality directly and negatively affected the state anxiety (*β* = −.41; *p* < .001) and pregnancy stress (*β* = −.36; *p* < .001). It also had a direct and positive significant impact on the coping domains including planning‐preparation (*β* = .36; *p* < .001), avoidance (*β* = .46; *p* < .001) and spiritual‐positive coping (*β* = .48; *p* < .001). Health promotion behaviours were negatively related to state anxiety (*β* = −.36; *p* < .001), but not to pregnancy stress (*β* = −.09; *p* = .14). Finally, avoidance (*β* = .07; *p* = .33) and spiritual‐positive coping (*β* = .08; *p* = .23) were not relevant to health promotion behaviours. However, planning‐preparation coping was significantly correlated to health promotion behaviours (*β* = .23; *p* = .001).

**TABLE 4:** Direct, indirect effects of latent variables (constructs) in conceptual model

Effect	Direct	*p*‐value	VIF
SWBS ‐> HPLP			
Direct	0.13	.04	1.54
Indirect	0.33	<.001	
Total	0.46		
SWBS ‐> State anxiety	−0.41	<.001	1
SWBS ‐> NuPDQ^a^	−0.36	<.001	1
SWBS ‐> planning‐preparation	0.36	<.001	1
SWBS ‐> avoidance	0.46	<.001	1
SWBS ‐> spiritual‐positive cop	0.48	<.001	1
State anxiety ‐> HPLP	−0.36	<.001	1.49
NuPDQ ‐> HPLP	−0.09	.14	1.38
planning‐preparation ‐> HPLP	0.23	.001	1.82
Avoidance ‐> HPLP	0.07	.33	1.95
spiritual‐positive cop ‐> HPLP	0.08	.23	1.70

Abbreviations: HPLP, Health Promoting Lifestyle Profile; SWBS, Spiritual Well‐Being Scale; VIF, Variance Inflammation Factor.

^a^ Revised Prenatal Distress Questionnaire.

**FIGURE 2:**
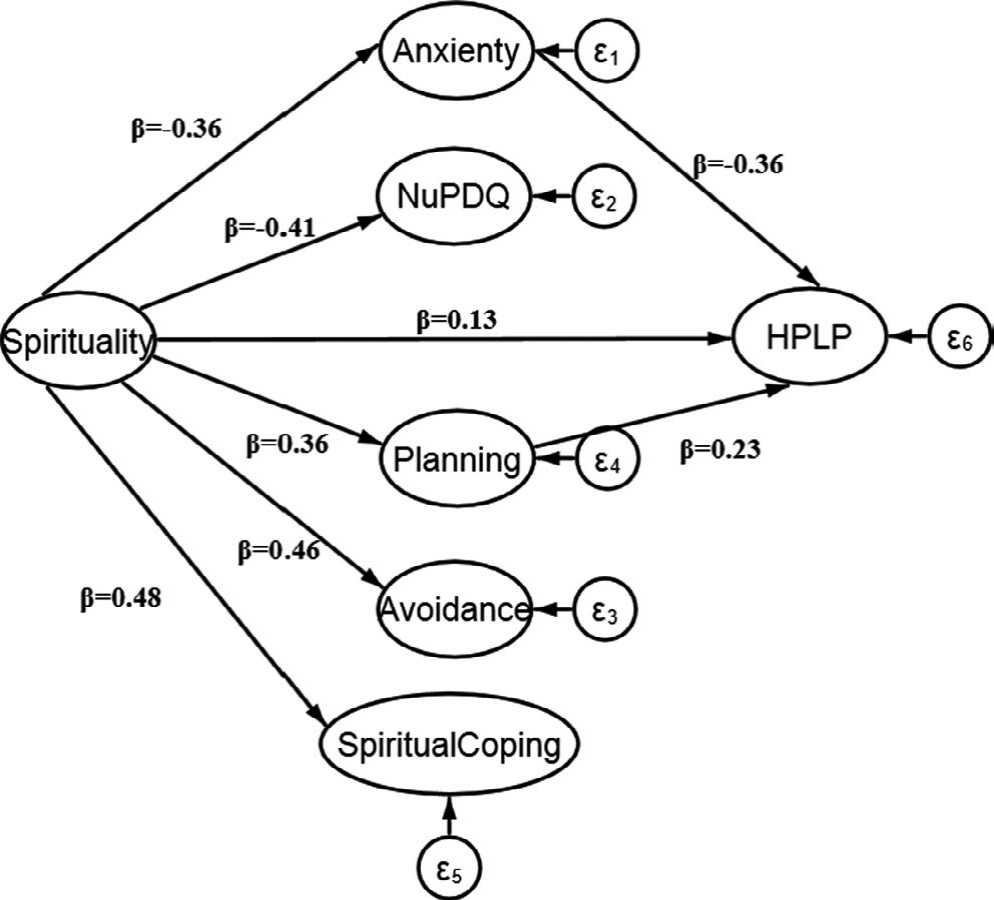
Final model of path diagram including significant coefficients for spirituality, prenatal Coping and Health Promoting Lifestyle Profile (HPLP)

Finally, PLS‐SEM analysis revealed a significant indirect effect of spirituality health on health promotion behaviours (indirect effect = 0.33; *p* < .001), mediated through its association with state anxiety and planning‐preparation coping (Figure 2).

## 4 DISCUSSION

The current study determined the effect of spiritually well‐being on health promotion behaviours of pregnant women in mediating the relationship between pregnancy stress, general anxiety, as well as coping ways and health promotion behaviours.

The results revealed the direct positive effect of spiritual well‐being on health promotion behaviours. In line with our results, a previous study reported that increasing religiosity was associated with reduced likelihood of smoking, alcohol use and drug abuse, as well as greater likelihood of better maternal nutrition during pregnancy (Burdette et al., 2012). A study reported that higher levels of religiosity were associated with more frequent health promotion behaviours (Cyphers et al., 2017). However, this study had a different measurement criterion compared to ours: the criterion measured in that study was religion index and religion commitment, while that of this study was spiritual well‐being.

Correlational findings showed that spiritual well‐being was negatively related with state anxiety and pregnancy stress. Our data support the conclusion that the main factor showing a negative effect on health promotion behaviours was state anxiety. Evidence has shown that spiritual well‐being predicts more than one‐third of state anxiety in adults (Steiner et al., 2017). Also, a study reported that spiritual well‐being was negatively correlated with pregnancy‐specific stress (Dolatian et al., 2017). A study reported that spirituality was associated with diminished anxiety of pregnant women (Mann et al., 2008). However, that study included pregnant women with– moderate‐to‐severe anxiety. In addition, the assessment of anxiety symptoms was based on the anxiety subscale of the Hospital Anxiety and Depression Scale (HADS). On the other hand, spirituality may be positively related to anxiety. A review study explained that spirituality can increase anxiety by means of negative beliefs, negative religious copings, misunderstanding and miscommunication (Weber & Pargament, 2014).

Another correlational finding was the positive correlation of spiritual well‐being with three types of pregnancy coping ways: planning‐preparation, avoidance and spiritual‐positive coping. We conclude that the effect of spiritual well‐being on coping ways and health promotion behaviours depends on the type of coping ways. Further, spiritual well‐being had a positive relationship with three kinds of copings. However, only planning‐preparation coping promoted the healthy behaviours, while the avoidance and spiritual‐positive coping did not have a significant effect on health promotion behaviours. A previous study indicated that spiritual coping was positively correlated with problem‐solving strategies and emotional‐solving in pregnant women (Faramarzi et al., 2017).

The results supported the indirect strong positive effect of spiritual well‐being on health promotion behaviours of pregnant women through the mediatory role of anxiety and planning‐preparation coping. Now, the question is how the planning‐preparation coping and state anxiety could mediate the relationship between spiritual well‐being and health promotion behaviours. The mechanism of effect of spirituality on anxiety or planning‐preparation coping as well as the impact of the mediatory effects on healthy behaviours is unknown. However, there are assumptions on the interpretation of the effects. First, the anxiety level is related to both spirituality and healthy behaviours. Research has shown that spirituality is associated with lower levels of anxiety and less perceived stress (Shattuck & Muehlenbein, 2020). Also, state anxiety could negatively predict healthy behaviours of pregnant women including healthy nutrition, physical activity and health responsibility (Omidvar et al., 2018). Secondly, spirituality helps pregnant women have greater well‐being. Individuals with higher spirituality can better cope with a stressful life and can have a sense of control as well as greater hope (Vasegh et al., 2012). Individuals with higher spirituality have higher social support and less social anxiety (Faramarzi & Pasha, 2015; Silton et al., 2014). Finally, the spiritual well‐being may improve the healthy behaviours of pregnant women by enhancing the planning‐preparation copings. It seems that the spirituality helps women increase their preparation to planning ways to cope with stressful (Faramarzi et al., 2016). Evidence supports that spirituality is strongly correlated with problem‐solving strategies in pregnant women (Faramarzi et al., 2017).

These findings underline the importance of anxiety and coping planning in mediating processes that explain how spiritual well‐being exerts its effects on health promotion behaviours of pregnant women. Further, investigating the association between mental disorders and health behaviours of pregnant women is recommended. Also, further research is necessary to determine the effect of spirituality on healthy behaviours for the maternal and neonatal pregnancy outcomes. Research should also explore how the interaction of spiritual well‐being and stress coping strategies influences the healthy behaviours of pregnant women; How avoidance coping would reduce the stress anxiety while leaving healthy behaviours unaffected; Could the healthy behaviours of pregnant women be enhanced through decreasing anxiety and increasing planning‐coping ways?

These findings can propose the path for various practical implications. Healthcare professionals, especially nurses and midwives, could emphasize the positive effects of spiritual well‐being on decreasing anxiety and pregnancy stress as well as promoting healthy behaviours. Educating mothers and their partners about the benefits of spiritual well‐being in decreasing anxiety and increasing planning‐copings may be an important facilitating factor for improving health promotion behaviours during the pregnancy period.

This study had some limitations which may restrict generalization. First, the design of the project was cross‐sectional; thus, cause‐effect conclusions should be drawn with caution. Cohort studies are required to assess the effect of spiritual well‐being on healthy behaviours of pregnant women. Secondly, the present findings were based on self‐reporting of the participants, thus the response bias may compromise the accuracy of results.

## 5 CONCLUSION

Spiritual well‐being may reduce state anxiety and pregnancy stress, while increasing planning‐preparation, avoidance and spiritual‐positive copings. Planning‐preparation coping and state anxiety mediated the relationship of spiritual well‐being with health promotion behaviours. This is a new fact that nurses or midwives may improve promotion of healthy behaviours directly by educating pregnant women about spiritual well‐being or indirectly by promoting planning‐preparation copings and reducing anxiety.

## CONFLICT OF INTEREST

The authors have declared no conflict of interest.

## AUTHOR CONTRIBUTIONS

MF contributed to the conception and design of the study. MCH and MF wrote the first draft of the paper. MF and SA and ME and SS revised the manuscript. ME and SA and SS help gathering the data. MCH have analysed the research data. All authors read and approved the final manuscript.

## Data Availability

The datasets used and/or analysed during the current study are available from the corresponding author on reasonable request.
